# Estimation and update of betweenness centrality with progressive algorithm and shortest paths approximation

**DOI:** 10.1038/s41598-023-44392-0

**Published:** 2023-10-10

**Authors:** Nan Xiang, Qilin Wang, Mingwei You

**Affiliations:** 1 Liangjiang International College, Chongqing University of Technology, Chongqing, 401135 China; 2 College of Computer Science, Chongqing University, Chongqing, 400044 China; 3 Chongqing Jialing Special Equipment Co., Ltd., Chongqing, 400032 China

**Keywords:** Computer science, Information technology, Scientific data, Information theory and computation

## Abstract

Betweenness centrality is one of the key measures of the node importance in a network. However, it is computationally intractable to calculate the exact betweenness centrality of nodes in large-scale networks. To solve this problem, we present an efficient CBCA (Centroids based Betweenness Centrality Approximation) algorithm based on progressive sampling and shortest paths approximation. Our algorithm firstly approximates the shortest paths by generating the network centroids according to the adjacency information entropy of the nodes; then constructs an efficient error estimator using the Monte Carlo Empirical Rademacher averages to determine the sample size which can achieve a balance with accuracy; finally, we present a novel centroid updating strategy based on network density and clustering coefficient, which can effectively reduce the computation burden of updating shortest paths in dynamic networks. The experimental results show that our CBCA algorithm can efficiently output high-quality approximations of the betweenness centrality of a node in large-scale complex networks.

## Introduction

Network analysis^1^ is a technique to investigate the structure and properties of networks, and one of the important tasks is to calculate the centrality of a node in the network^2–4^, which measures how connected or influential a node is within the network. Common centrality measures are degree centrality^5^, betweenness centrality^6^, closeness centrality^7^, etc. The betweenness centrality of a node has many applications in various domains, such as identifying critical nodes in transportation networks^8^, detecting essential proteins in protein networks^9^, and improving clustering^10^ and community detection algorithms^11^.

Betweenness centrality (BC) measures the importance of a vertex or an edge based on the shortest paths in a graph (i.e., a vertex or an edge with higher BC appears more frequently on the shortest paths in the graph). Several exact algorithms for computing Betweenness centrality have been proposed^12–16^, among which Brandes’ algorithm^12^ is a representative one that uses the single source shortest paths (SSSP) idea to optimize the computation process. The time complexity of this algorithm is $O(nm+n^2log_n)$ for weighted graphs and *O*(*nm*) for unweighted graphs, where *n* and *m* are the numbers of vertices and edges in the graph, respectively.

However, exact algorithms are infeasible for large-scale networks^17^ due to their increasing size, and research emphasizes the ordering of nodes over exact values. Hence, some sampling-based approximation algorithms have emerged^18–30^, which can generate a betweenness centrality approximation with a high probability $(1-\delta )$ and a bounded maximum deviation, satisfying $\varepsilon -approximation$^31^. However, this category of approximation algorithms faces some challenges, such as determining the sample size that can represent the global distribution of parameters with the least samples; selecting samples that can better estimate the parameter distribution; and adapting to changes in network dynamics. Currently, some researchers have proposed many attempts to address these problems. For example, Brandes^29^ proposed a *bp* algorithm based on Hoeffding’s inequality and union bound, but this algorithm relies too much on the number of nodes *n*, resulting in excessive running overhead; then Matteo et al^22^ proposed an *rk* algorithm based on Vapnik-Chervonenkis (VC) dimension theory, using VC dimension theory to limit the sample size so that the sample no longer depends on the number of nodes in the graph but on the diameter of the graph (i.e., the number of nodes with the largest shortest path in the graph). Nonetheless, the VC dimension does not yield the best $\varepsilon -approximation$ for a given number of $\delta$; therefore, Matteo et al^20^ proposed another *ab* algorithm based on the Rademacher averages^32^ and progressive sampling^33^ to estimate the betweenness centrality of the nodes. Rademacher averages with probability bounds^34–36^ and VC dimensions are also often used in pattern mining^37–39^. Bergamini et al^40^ proposed a new fully dynamic adaptive sampling update algorithm based on Matteo’s shortest paths sampling.

At the same time, the network is dynamic, with the addition or deletion of nodes and edges at any time. It would be costly to recalculate the betweenness centrality for each dynamic change. Therefore, many dynamic updating algorithms have been proposed^41–49^. These algorithms store the nodes or edges that change by using data structures to avoid re-computing the betweenness centrality from scratch. Hayashi^41^ proposed a fully dynamic algorithm and a new technique called Two-ball-index and Special-Purpose Reachability Index on the hypersketch sketch of Yoshida^26^, which improved the dynamic update by several orders of magnitude compared to Berigamini^48^. Berigamini extensively updated the number of pairwise distances and shortest paths and proposed a faster algorithm to update dependencies^40^. This algorithm decreased the number of operations performed by dynamic betweenness centrality algorithms, but they have the same worst-case running time as recalculations.

Although the above methods propose many improvement strategies for estimating the betweenness centrality, they do not improve the sampling method and consider network characteristics. To address the above problems, we present an efficient approximation algorithm CBCA (Centroids-based Betweenness Centrality Approximation) based on progressive sampling and shortest paths approximation. The algorithm firstly approximates the shortest paths by using the adjacency information entropy of nodes to generate the network centroid; then constructs Monte Carlo Empirical Rademacher averages based estimator to determine the sample size and achieve a balance between accuracy and efficiency; finally, we propose a new centroid updating strategy according to the network density and clustering coefficients, which effectively reduces the cost of updating the shortest paths in dynamic networks.

The main contributions and innovations in this paper are as follows:

- We present a novel error estimator based on Monte Carlo Empirical Rademacher Averages (MCERA) and progressive sampling methods, which can balance the trade-off between sample size and accuracy. By using MCERA and adjusting the sample size according to the accuracy requirement, our estimator can effectively guarantee that the upper probability bound of the approximation error of the CBCA algorithm is within a predefined range. Experimental results demonstrate that the CBCA algorithm with our estimator is faster and requires smaller samples than existing methods under the same probability condition.
- We propose a strategy to update the centroid in dynamic networks with frequent BC changes. Our strategy uses a dynamic detection mechanism that monitors the network density and clustering coefficients, which are important features of the network graph. The mechanism updates the centroid when these features exceed a set threshold, without adding or deleting nodes and edges at any time. This reduces the time to reselect high-quality centroids in dynamic networks.
- We propose an improved shortest paths approximation method based on network centroids for sampling. Our method can be applied to both undirected and directed graphs. To select the number of centroids, we use different calculation methods of diameter and reduce the computation of dynamic network shortest paths update by distinguishing between undirected and directed graphs. We use the adjacency information entropy^50^ to select high quality centroids. The experimental results show that our method can cover more than 80% of the shortest paths in networks with scale-free^51^ and small-world characteristics^52^.

## Preliminaries

We will describe the definitions and basic ideas in this section.

### Graphs and betweenness centrality

Let $G=(V,E)$ be a graph, either undirected or directed, and where each edge must have a non-negative weight. We denote $n=|V|$ in the graph, and *n* is the number of all nodes. For any distinct node pair (*u*, *v*) in the graph, where $u \ne v$. Let $\sigma _{uv}$ denote the number of all shortest paths between node *u* and node *v*, and let $\sigma _{uv}(w)$ be the number of shortest paths between node *u* and node *v* for all shortest paths passing through *w*. For convenience, we write the shortest paths as *SPs*.

Given a graph $G=(V,E)$, the normalized betweenness centrality *b*(*w*) of a node $w \in V$ is defined as:

1 $$\begin{aligned} \begin{aligned} b(w)&= \frac{1}{n(n-1)}\sum _{(u,v)\in V \times V,u \ne v}\frac{\sigma _{uv}(w)}{\sigma _{uv}}. \end{aligned} \end{aligned}$$

### Rademacher averages

Rademacher averages^36^ are an essential core of statistical learning theory^53^ and allow measuring the convergence speed of sample means concerning their expectations. More detailed information on the Rademacher averages can be found in^35,36^.

Define a finite field *P* and a uniform distribution $\mu$ over the elements from *P*. Let ${\mathscr {F}}$ be a family of functions from *P* to [0,1], and let $S=\{S_1,...,S_m\}$ be the set of *m* independent identically distributed samples of *P* with probability uniform distribution $\mu$. The average value and its expectation for each function *f*, which is defined over the samples *S*, are as followed, respectively:

2 $$\begin{aligned} \begin{aligned} \rho _s(f)&= \frac{1}{m}\sum _{i=1}^m f(s_i)\\ \rho _{\mu }(f)&= E[\rho _s(f)] \,, \end{aligned} \end{aligned}$$

i.e., $\rho _s(f)$ is an unbiased estimator of $\rho _{\mu }(f)$.

Now, for a given *S*, we focus on the upper bound of the supremum deviation $S({\mathscr {F}},S)$ of $\rho _s(f)$ from $\rho _{\mu }(f)$ among all $f \in {\mathscr {F}}$, the quantity is:

3 $$\begin{aligned} \begin{aligned} S({\mathscr {F}},S) = \mathop {sup}\limits _{f \in {\mathscr {F}}}|\rho _s(f)-\rho _{\mu }(f)| \,, \end{aligned} \end{aligned}$$

The $S({\mathscr {F}},S)$ is the vital notion in the study of empirical processes. The Empirical Rademacher Averages (ERA) $\tilde{R}({\mathscr {F}},S)$ of ${\mathscr {F}}$ on *S* is the indicator that can effectively determine the sample-dependent upper bound of the supremum deviation $S({\mathscr {F}},S)$, which can take into account the data distribution relationship. Let $\lambda = (\lambda _1,..., \lambda _m)$ is the collection of *m* independent identically distributed (i.e., i.i.d) Rademacher random variables that takes the values $\{-1,1\}$ with the same probability $\frac{1}{2}$ of taking -1 or 1, respectively. This quantity is:

4 $$\begin{aligned} \begin{aligned} \tilde{R}({\mathscr {F}},S)=E_{\lambda }\bigg [\mathop {sup}\limits _{f \in {\mathscr {F}}}\frac{1}{m}\sum _{i=1}^m \lambda _i f(s_i)\bigg ]. \end{aligned} \end{aligned}$$

However, $\tilde{R}({\mathscr {F}},S)$ is difficult and costly to compute. Monte Carlo estimation^35^ gives an efficient way to get the apparent sharply probability bound for ERA. For any $k \ge 1$, let $\lambda \in \{-1,+1\}^{k \times m}$ be a matrix of $k \times m$ that is independently identically distributed for the Rademacher random variables. The *k*-Trials Monto-Carlo Empirical Rademacher Averages (i.e., *k*-MCERA) $\tilde{R}_m^k({\mathscr {F}},S,\lambda )$ of ${\mathscr {F}}$ on *S* using $\lambda$ is:

5 $$\begin{aligned} \begin{aligned} \tilde{R}_m^k({\mathscr {F}},S,\lambda ) = \frac{1}{k} \sum _{j=1}^k \mathop {sup}\limits _{f \in {\mathscr {F}}}\frac{1}{m}\sum _{i=1}^m \lambda _{j,i}f(s_i)\,, \end{aligned} \end{aligned}$$

The Empirical Rademacher Averages $\tilde{R}({\mathscr {F}},S)$ is the expectation of *k*-Trials Monto-Carlo Empirical Rademacher Averages $\tilde{R}_m^k({\mathscr {F}},S,\lambda )$ of ${\mathscr {F}}$ on *S*, which controls the probability bound of $S({\mathscr {F}},S)$. It is not efficient to use the Rademacher averages to obtain clear probability bounds, but rather to use *k*-MCERA to make a better balance between sample size and accuracy. That is because *k*-MCERA can directly estimate the highest deviation of the function set by data dependence. For each $f \in {\mathscr {F}}$, We define the empirical wimpy variance as $\alpha$, defined as:

6 $$\begin{aligned} \begin{aligned} \alpha = \mathop {sup}\limits _{f \in {\mathscr {F}}}\frac{1}{m}\sum _{i=1}^m(f(s_i))^2. \end{aligned} \end{aligned}$$

Before stating Theorem 2.2, we need to know the upper bound of the wimpy variance (i.e., Theorem 2.1). Then, Theorem 2.2 shows how to use the *k*-MCERA to compute the upper bound of the maximum deviation $S({\mathscr {F}},S)$, using only sample-dependent quantities.

#### Theorem 2.1

*For any* $f \in {\mathscr {F}}$ *is a family of functions in the domain* *P* *up to [0,1] and* $\mu$ *is a uniform distribution of probabilities over the elements of* *P*:

7 $$\begin{aligned} \begin{aligned} var = \mathop {sup}\limits _{f \in {\mathscr {F}}}E_{\mu }[f^2]. \end{aligned} \end{aligned}$$

#### Proof

We have given in Online Appendix A.1, which leverages the properties of the betweenness centrality under large-scale networks and the basic variance formula.

#### Theorem 2.2

*For* $k,m \ge 1$ *and the function* $f \in {\mathscr {F}}$, *where* ${\mathscr {F}}$ *is a family of functions from* *P* *to [0,1]*. *Let* $\lambda \in \{-1, +1\}^{k \times m}$ *be a* $k \times m$ *matrix of Rademacher random variables, such that independently and with equal probability* $\frac{1}{2}$. Let *S* *be a sample size of* *m* *drawn i.i.d. from* *P*, *and take a distribution* $\mu$. *For each* $f \in {\mathscr {F}}$, $\delta \in (0,1)$, *define:*

8 $$\begin{aligned} \begin{aligned} V(f)&\doteq \alpha + \frac{ln\frac{3}{\delta }}{m} + \sqrt{\left( \frac{ln\frac{3}{\delta }}{m}\right) ^2 + \frac{2\alpha ln\frac{3}{\delta }}{m}} \\ \tilde{R}({\mathscr {F}},S)&\doteq \tilde{R}_m^k({\mathscr {F}},S,\lambda ) + \frac{2ln\frac{3}{\delta }}{km} + \sqrt{\left( \frac{2ln\frac{3}{\delta }}{km}\right) ^2 + \frac{4(\tilde{R}_m^k({\mathscr {F}},S,\lambda ) + \alpha )ln\frac{3}{\delta }}{km}} \\ R({\mathscr {F}},m)&\doteq \tilde{R}({\mathscr {F}},S) + \frac{ln\frac{3}{\delta }}{m} + \sqrt{\left( \frac{ln\frac{3}{\delta }}{m}\right) ^2 + \frac{2\tilde{R}({\mathscr {F}},S)ln\frac{3}{\delta }}{m}} \\ \varepsilon&\doteq 2R({\mathscr {F}},m) + \frac{ln\frac{3}{\delta }}{3m} + \sqrt{\left( \frac{ln\frac{3}{\delta }}{3m}\right) ^2 + \frac{2R({\mathscr {F}},m)ln\frac{3}{\delta }}{m}}\,, \end{aligned} \end{aligned}$$

*With the probability at least* $1 - \delta$ *over the choice of* *S* *and* $\lambda$, *it holds*:

9 $$\begin{aligned} \begin{aligned} S({\mathscr {F}},S) \le \varepsilon . \end{aligned} \end{aligned}$$

#### The proof of Theorem 2.2

We put in Online Appendix A.2, using the self-boundary function^54^ and the symmetry inequality^55^, as well as the substitution theorem.

Observing Theorem 2.2, we can see that $\alpha$ is the largest factor affecting the whole equation and controls the supremum deviation $S({\mathscr {F}},S)$. Thus, we can achieve a better balance between sample size and accuracy, obtaining a uniform variance for most families of functions. This is the reason why *k*-MCERA outperforms *rk* and *bp*. The *rk* algorithm uses VC dimensional theory to obtain an upper limit on the sample size, which is data-independent and depends on the properties of the graph itself. However, it does not consider the feature of data dependence. The *bp* algorithm uses Hoeffding’s inequality and union bound, which results in an excessive sample. These two methods lead to a large number of samples to guarantee a high quality approximation, so they are characterized by a sample size that is suboptimal, while *k*-MCERA can capture the relationship between sample size and accuracy very well.

### Network density and clustering coefficient

In this section, we focus on the dynamic changes of complex networks.

We know that dynamic networks change by adding or deleting nodes and edges, but this requires updating and maintenance. However, not all nodes or edges addition and deletion have a significant impact on the network, and we can ignore some minor changes while pursuing approximate estimates. According to^56–58^, network density and clustering coefficient are closely related to the power law property and small world property of complex networks, which are the key assumptions of our algorithm. Therefore, we choose the network density and clustering coefficient, which are two parameters that can reflect the features of the network. And the network is considered to have been changed measurably when their changes exceed a certain threshold value. The centroid is a critical factor that affects the efficiency of the whole algorithm.

We present a strategy to update the network centroid based on the network density and clustering coefficients to detect the dynamic changes, thus avoiding a large amount of updating time.

Network density^59^: it can be used to characterize the density of interconnected edges of nodes in a network, which is defined as the ratio of the number of variables present in the network with the upper limit of the number of edges that can be accommodated. It is commonly used to measure the intensity of social relationships and the evolutionary trend in online social networks. For $G=(V,E)$ with *n* nodes and *m* edges, the network density is defined as:

10 $$\begin{aligned} \begin{aligned} d_G&= \frac{2m}{n(n-1)}. \end{aligned} \end{aligned}$$

Clustering coefficient^60,61^: it quantifies how densely nodes form cliques in a graph. There is evidence^62^ that nodes tend to create tightly bound groups in most real-world networks, especially social networks. This is divided into a global clustering coefficient and a local clustering coefficient. We choose the local clustering coefficient as one of the parameters to reflect the features of the network, because it can capture the local structural changes of nodes and their neighbors in dynamic networks.

For $G=(V,E)$, where $V=(v_1,v_2,...,v_n)$ denotes the collection of vertices and $E=\{e_{ij}:(i,j) \in U \subset [1,...,n]^2\}$ denotes the collection of edges ($e_{ij}$ denotes the edge connecting vertices $v_i$ and $v_j$). Denote by *L*(*i*) the collection of edges connected to the vertex $v_i$: $L(i) = \{ v_j: e_{ij} \in E \wedge e_{ji} \in E \}, z_i$ is the degree of node *i*. The local clustering coefficient of vertex $v_i$ in an undirected graph is:

11 $$\begin{aligned} \begin{aligned} C(i)&= \frac{2|\{e_{jk}:v_j,v_k \in L(i), e_{jk} \in E\}|}{z_i(z_i-1)}. \end{aligned} \end{aligned}$$

The local clustering coefficient of vertex $v_i$ in a directed graph is:

12 $$\begin{aligned} \begin{aligned} C(i)&= \frac{|\{e_{jk}:v_j,v_k \in L(i), e_{jk} \in E\}|}{z_i(z_i-1)}. \end{aligned} \end{aligned}$$

The average clustering coefficient can be obtained as:

13 $$\begin{aligned} \begin{aligned} {\overline{C}}&= \frac{1}{n}\sum _{i=1}^n C(i). \end{aligned} \end{aligned}$$

To facilitate the reader’s understanding of the article, we provide the explanations of the main parameters in Table 1.

**Table 1:** Main Parameters.

Symbols	Definition
$G=(V,E)$	Networks
*V*	Vertexes
*E*	Edges
*t*	Centroids
*m*	Number of samples
*b*(*w*)	Betweenness centrality
$\varepsilon$	Absolute error
*n*	$n=|V|$, number of nodes
*k*	*k*-Monto-Carlo-Rademacher trials
$d(u,v) = d(u,t_1)+d(t_1,t_2)+d(t_2,v)$	Shortest path approximation
$\tilde{b}(w)$	$\varepsilon$-approximation of betweenness centrality
$\delta$	$P(\varepsilon -approximation of b(w)) \ge 1-\delta$
$\sigma _{uv}$	The number of all SPs between node *u* and node *v*.
$\sigma _{uv}(w)$	The number of SPs between node *u* and node *v* for all SPs via *w*.

## Methods

This section shows our proposed CBCA algorithm, which is an efficient approximation algorithm based on progressive sampling and shortest paths approximation.

Progressive sampling is a technique that gradually increases the sample size until a desired accuracy is achieved. It allows us to avoid over-sampling or under-sampling the network, and adapts to the dynamic changes of the network. Shortest paths approximation is a technique that uses the network centroids to estimate the shortest paths between nodes. It allows us to reduce the computational complexity and memory requirement of the algorithm.

We firstly introduce the basic process and related results for approximating the shortest paths based on the network centroids, which can efficiently compute the betweenness centrality values after approximating the shortest paths in section “Shortest paths approximation based on network centroids”, and we have provided the data-dependent bounds for these results in Theorem 2.2. Then, we describe the specific steps and parameters of the CBCA algorithm in Section 3.2, which uses this improved bound to obtain high-quality betweenness centrality approximations with high probability and ensures that the betweenness centrality values of all nodes are within the additive error $\varepsilon$ by progressive sampling. The detailed theoretical and experimental results regarding the initial sample size, the selection of the sample schedule, and the sections on updating the network centroids are given in the introduction section of Experiment 4 and Experiment 4.5, respectively.

### Shortest paths approximation based on network centroids

The common shortest paths algorithms do not exploit the properties of complex networks (i.e., scale-free network characteristic (power-law property) and small-world property). We leverage these two properties to improve a shortest paths approximation method based on the network centroids. The small-world network has two important properties: high clustering coefficient and short average shortest paths length. The scale-free network characteristic means that few nodes have a high degree and most nodes have a low degree. Then we can naturally select some nodes with high degree or high adjacency information entropy as the centroids, and divide the nodes near or adjacent to the centroids into subgroups. From these subgroups, we use the shortest paths algorithm to calculate the distance between each node and the centroids. This can reduce the search space and time. And it can improve the approximation accuracy. Therefore, the number and quality of the centroids are crucial.

#### Centroids screening strategy

We use the VC dimension theory to calculate the number of *t* centroids in statistical learning, and we refer the reader to^32^ for more details on the VC dimension theory:

14 $$\begin{aligned} \begin{aligned} D&= max|SP_s|\\ t&= c\Bigg (\lfloor log_2(D-2)\rfloor +1+ln\frac{1}{\delta }\Bigg )\,, \end{aligned} \end{aligned}$$

where *t* is the number of centroids with *c* taking the value $\frac{1}{3}$, *D* is the diameter of the graph, and $\delta \in (0,1)$.

The equation is derived from Matteo^22^, but *c* is not the meaning of the coefficients among them in this paper, and they are irrelevant. The reason for employing this equation is that with the data independence of the VC dimension, where the number of centroids does not depend on the number of nodes, but only on the diameter in the graph (i.e., the maximum length of the shortest path). It reduces the number of *t* and speeds up the approximation time. At the same time, the value of *c* can affect that of *t*. We need to trade-off between the number of centroids and the ratio $\frac{T_1}{T_0}$ ($T_1$ is the shortest paths after approximation and $T_0$ is the shortest paths without approximation) to get an acceptable value. According to experiments, we found that the quality of the centroids is best when $c=\frac{1}{3}$ and $t=2 or 3$. This range of values facilitates the calculation of the shortest paths approximation.

Furthermore, in this Eq. (14), we need to know the diameter of the graph *D*. One way to compute the exact value of *D* is by solving the shortest paths problem between all pairs of nodes. However, this exact calculation of the diameter *D* is not desirable, because it requires a time complexity of $O(n^3)$, which is against our experimental purpose. Given the short average path length of complex networks and the data independent property of VC dimension, we can reason that the error of diameter has little or even negligible effect on the result. Thus, we adopt different approximate diameter calculation methods for directed and undirected graphs respectively, in this paper.

Approximate diameter methods:

1. Let $G=(V,E)$ be an undirected graph with all edges having equal weights. Choosing a vertex $u \in V$ uniformly and randomly from the graph and computing the shortest paths from *u* as the source to all other nodes. We can calculate the diameter *D* equal to the sum of the two shortest paths from *u* to two other different nodes *w*, *v*.

2. For a directed graph with weights, we note that *D* is not necessarily equal to the longest path among the shortest paths between all pairs of nodes, because the edge weights may affect the distance sums. This makes the calculation of the approximate diameter more complicated. We can use the maximum weakly connected component, as an upper bound on the diameter *D*.

#### Centroids quality screening strategy

We use the adjacency information entropy formula to effectively filter out high-quality centroids based on the small-world feature and the scale-free characteristic of complex network. Commonly used information entropy formulas are^50,63,64^. In this paper, we choose a more popular adjacency information entropy formula^50^ and give a reasonable explanation.

##### Definition 1

For a given *G*(*V*, *E*), where $V(v_1,v_2,...,v_{n-1},v_n)$ refers to all nodes and $E(e_1,e_2,...,e_{l-1},e_l)$ refers to all edges.

The undirected unweighted graph *G*(*V*, *E*): The node degree of the undirected unweighted graph can be obtained by $h_i=\sum \limits _1^l o_{ij}$, where *l* is the number of node *i* and *j* is the neighbor of node *i*. $o_{ij}$ is equal to 1 if an edge exists between node *i* and node *j*, otherwise it is 0.

The undirected weighted graph *G*(*V*, *E*): The node degree of the undirected unweighted graph can be obtained by $h_i=\sum \limits _1^l w_{ij}$, where *l* is the number of node *i*, *j* is the neighbor of node *i*, and $w_{ij}$ represents the weight magnitude between node *i* and node *j*.

The degrees in the directed graph are divided into in degrees and out degrees, which are divided into two cases, directed with weights and directed without weights, as follows.

Directed unweighted graph *G*(*V*, *E*):

15 $$\begin{aligned} \begin{aligned} h_i^{in}&= \sum _{j \in \xi _i}o_{ji}\\ h_i^{out}&= \sum _{j \in \xi _i}o_{ij}\,, \end{aligned} \end{aligned}$$

Directed weighted graph *G*(*V*, *E*):

16 $$\begin{aligned} \begin{aligned} h_i^{in}&= \sum _{j \in \xi _i}w_{ji}\\ h_i^{out}&= \sum _{j \in \xi _i}w_{ij}\,, \end{aligned} \end{aligned}$$

Where $\xi _i$ is the set of the neighbors of node *i*.

When discussing the influence of graphs, we need to distinguish between directed and undirected graphs. In a directed graph, each node has an incoming degree and an outgoing degree, which indicates the number of edges pointing to and from that node, respectively. In an undirected graph, each node has a degree, which indicates the number of edges connected to that node. Thus, we introduce an influence factor $\zeta$ to measure the influence of a node. It is a constant between 0 and 1 to regulate the contribution of incoming and outgoing degrees to influence. Usually, $\zeta$ taken as 0.7 is a reasonable choice. In an unweighted graph, the calculation of influence is relatively simple by multiplying the in degree of nodes by $\zeta$ and adding the out degree by $1-\zeta$. In the weighted graph, the calculation of influence is more complicated, which requires considering the weights and directions of the edges, as follows:

Directed unweighted graph *G*(*V*, *E*):

17 $$\begin{aligned} \begin{aligned} h_i^{unweighted}&= \zeta h_i^{in} + (1-\zeta )h_i^{out} = \zeta \sum _{j=1}^l o_{ji} + (1-\zeta ) \sum _{j=1}^l o_{ij}\,, \end{aligned} \end{aligned}$$

Directed weighted graph *G*(*V*, *E*):

18 $$\begin{aligned} \begin{aligned} h_i^{weighted}&= \zeta h_i^{in} + (1-\zeta )h_i^{out} = \zeta \sum _{j=1}^l w_{ji} + (1-\zeta ) \sum _{j=1}^l w_{ij}. \end{aligned} \end{aligned}$$

##### Definition 2

Adjacency $$A_i$$:

19 $$\begin{aligned} \begin{aligned} A_i&= \zeta \sum _{j \in \xi _i}k_{j_{in}} + (1-\zeta ) \sum _{j \in \xi _i}k_{j_{out}}\,, \end{aligned} \end{aligned}$$

we can easily obtain that $A_i=\sum \limits _{j \in \xi _i}k_j$, if the graph is undirected.

##### Definition 3

Probability $P_{i_j}$, we define the selection probability of node *i* in the network by considering the probability of node *i* being selected by its neighbor *j* with the following probability:

20 $$\begin{aligned} \begin{aligned} P_{i_j}=\frac{k_i}{A_j},(j \in \xi _i). \end{aligned} \end{aligned}$$

##### Definition 4

Information entropy formula, the important technical formula is used to carry out how to filter *t* network centroids to achieve the properties of complex networks in this paper, which compound our assumptions:

21 $$\begin{aligned} \begin{aligned} E_i&= - \sum _{j \in \xi _i} (P_{i_j}log_2P_{i_j}) \quad if\ the\ graph\ is\ undirected \\ E_i&= \sum _{j \in \xi _i} |-P_{i_j}log_2P_{i_j}| \quad if\ the\ graph\ is\ directed\,, \end{aligned} \end{aligned}$$

by the above way, we can reasonably screen out the high-quality centroids by the adjacency information entropy.

#### Subgraphs construction for the shortest paths approximation

In this section, we describe in detail how to generate subgraphs and how centroids of prime nodes are generated. As an example of an undirected unweighted graph, the same method is used for the other types of graphs.

*Step 1*

**Figure 1:**
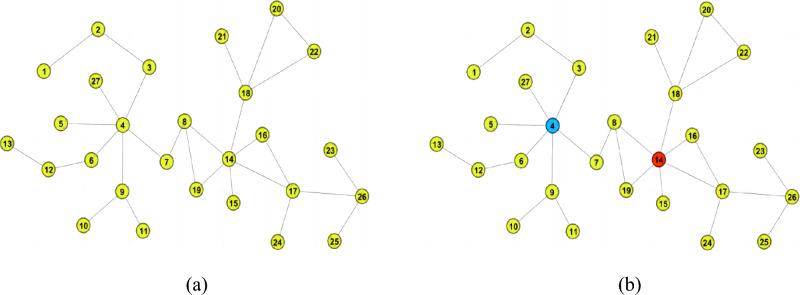
(**a**) Initial input graph. (**b**) The required number of centroids calculated from the diameter and the adjacency information entropy is used to screen the high-quality centroids.

We use the adjacency list to represent the graph structure, so one Breadth-First Search (BFS) traversal gives us the degrees and weights of all nodes. As shown in Fig. 1a.

*Step 2*

When performing the BFS traversal, we get the diameter by the approximate diameter calculation method in section "Centroids screening strategy", (i.e., D = 10.) In addition, we use the method of screening the number and quality of centroids mentioned in sections "Centroids screening strategy" and "Centroids quality screening strategy". The method of screening the number of centroids is based on the diameter of the graph and a given confidence parameter $\delta$ to determine the number of centroids to be selected as *t*. The formula is $t=c(\lfloor log_2(D-2) \rfloor + 1 + ln \frac{1}{\delta })$, where $c=\frac{1}{3}, \delta =0.1$. We get $t=2.1$, so there are two centroids. Then we utilize the adjacency information entropy formula to easily get the two high-quality centroid nodes, as shown in Fig. 1b).

*Step 3*

**Figure 2:**
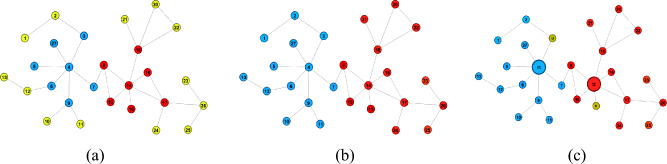
(**a**) First traversal of the nearby neighboring node. (**b**) Complete all subgraphs division states. (**c**) Shortest paths approximation between the centroids and node pairs in the subgraphs state.

The nodes surrounding the centroids can be easily obtained by traversing the neighboring nodes one at a time, as in Fig. 2a. Then continue to traverse the neighboring nodes and finally a complete subgraph can be obtained as shown in Fig. 2b.

Eventually, we get the complete graph with two centroids by the above method.

We explain in detail how to perform the shortest paths approximation. According to section “Shortest paths approximation based on network centroids”, we can reasonably infer that most of the shortest paths pass through the centroids of the network. Eqs. (22) and (23) can effectively reduce the influence of paths that do not pass through the centroids on sampling and estimation of the betweenness centrality. The experimental section “Different types of networks” verifies our theoretical hypothesis.

As shown in Fig. 2c, the shortest paths after approximation becomes $d(u,v) \doteq d(u,t_1)+d(t_1,t_2)+d(t_2,v)$.

Let the relationship of node pairs be written as $\delta _{uv}(w)=\frac{\sigma _{uv}(w)}{\sigma _{uv}}$:

22 $$\begin{aligned} \begin{aligned} \sigma _{uv}&= \sigma _{ut_1} \times \sigma _{t_1t_2} \times \sigma _{t_2v}\\ \sigma _{uv}(w)&= \sigma _{ut_1}(w) \times \sigma _{t_1t_2} \times \sigma _{t_2v}+\sigma _{ut_1} \times \sigma _{t_1t_2}(w) \times \sigma _{t_2v}+\sigma _{ut_1} \times \sigma _{t_1t_2} \times \sigma _{t_2v}(w). \end{aligned} \end{aligned}$$

Then we can have:

23 $$\begin{aligned} \begin{aligned} b(w)&= \frac{1}{n(n-1)}\sum _{(u,v)\in V \times V, u \ne v}\frac{\sigma _{uv}(w)}{\sigma _{uv}} \\&= \frac{1}{n(n-1)}\sum _{(u,v)\in V \times V, u \ne v}(\delta _{ut_1}(w)+\delta _{t_1t_2}(w)+\delta _{t_2v}(w)). \end{aligned} \end{aligned}$$

### CBCA algorithm description and analysis

In this section, we present the CBCA algorithm, which is based on the contributions of section “Shortest paths approximation based on network centroids”, for computing a strict approximation of the betweenness centrality of all nodes in the graph. We firstly describe the effective estimators that can satisfy the $\varepsilon - approximation$, which is an important component of the CBCA algorithm, for estimating the betweenness centrality in section “Effective estimator description and analysis”. Then we describe the CBCA algorithm in section “Our CBCA algorithm flow”.

#### Effective estimator description and analysis

Our CBCA algorithm takes as input a graph $G=(V,E)$, which can be directed or undirected and can have non-negative weights on the edges and includes two parameters $\varepsilon , \delta \in (0,1)$. It can output a set $\tilde{B}=\{\tilde{b}(w),w \in V\}$ (i.e., with probability at least $1-\delta$, betweenness centrality $B=\{b(w),w \in V of \varepsilon approximation\}$). Let $P=\{(u,v) \in V \times V, u \ne v\}$ be the collection of all different node pairs. For each node $w \in V$, let $f_w$: the function of *P* mapping to [0,1].

To improve the computational efficiency, we use:

24 $$\begin{aligned} \begin{aligned} f_w(u,v)&= \delta _{uv}(w)=\delta _{ut_i}(w)+\delta _{t_it_j}(w)+\delta _{t_jv}(w)\\&= f_w(u,t_i)+f_w(t_i,t_j)+f_w(t_j,v)\,, \end{aligned} \end{aligned}$$

let the set $S=\{(u_i,v_i)=(u_i,t_i)+(t_i,t_j)+(t_j,v_i), 1 \le i \le m\}$, where $t_i,t_j$ refer to the centroids in $u_i,u_j$, respectively, from the set of independent and uniformly sampled node pairs (*u*, *v*) from *P*. We define:

25 $$\begin{aligned} \begin{aligned} \tilde{b}(w)=\rho _s(f_w)=\rho _s(f_w(u_i,v_i))=\frac{1}{m}\sum _{i=1}^m f_w(u_i,v_i)=\frac{1}{m}\sum _{i=1}^m \frac{\sigma _{u_iv_i}(w)}{\sigma _{u_iv_i}}. \end{aligned} \end{aligned}$$

Thus, we have that

26 $$\begin{aligned} \begin{aligned} \rho _{\mu }(f)=E(\rho _s(f))=\frac{1}{n(n-1)}\sum _{(u,v) \in V \times V, u \ne v}\frac{\sigma _{uv}(w)}{\sigma _{uv}}=b(w). \end{aligned} \end{aligned}$$

#### Our CBCA algorithm flow

Our CBCA algorithm is a method based on a progressive algorithm. A progressive algorithm is an algorithm that checks whether a certain stopping condition is satisfied after each iteration. If it is satisfied, the final result is output; if not, the iteration continues. The goal of the CBCA algorithm is to estimate an approximation to the betweenness centrality *b*(*w*) of each node *w* in the graph and output an approximate set $\tilde{B}=\{\tilde{b}(w), w \in v\}$. To improve efficiency, the CBCA algorithm does not iterate through all possible node pairs, but samples node pairs from the set $S_i$, which is used to estimate *b*(*w*). Here $m_i=|S_i|$ denotes the size of the set $S_i$. Therefore, it is important to choose a suitable stopping condition, which affects the accuracy and speed of the CBCA algorithm. Especially for large graphs, the computational cost of each sample is high.

We now present the CBCA algorithm and next show how to obtain the set of $B=\{b(v), v \in V\}$ for $\varepsilon -approximation$.

**Algorithm 1:**
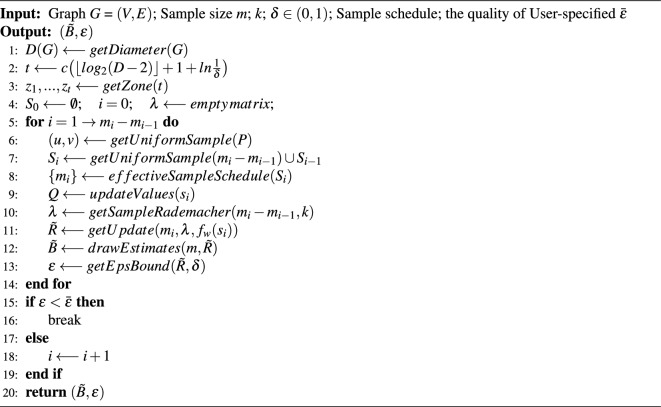
CBCA

The input parameters of the CBCA algorithm: a graph, a failure probability $\delta \in (0,1)$, the number of *k* for the *k*-MCERA, a user-specified error ${\bar{\varepsilon }}$, sample schedule, and a suitable sample size $m \>0$. The output is a pair $(\tilde{B},\varepsilon )$, where $\tilde{B}$ is a set of pairs $(v,\tilde{b}(v))$ for each $v \in V$, where $\tilde{b}(v)$ is the estimate of *b*(*v*), and $\varepsilon \in (0,1)$ is the accuracy that is probabilistically guaranteed in the following Theorem 3.1.

##### Theorem 3.1

*With probability at least* $1-\delta$ *for the CBCA algorithm*, *the output* $(\tilde{B},\varepsilon )$ *such that* $|b(v)-\tilde{b}(v)| \le \varepsilon$.

This is presented in Online Appendix A.3, using the converse method.

Our CBCA algorithm computes an $\varepsilon -approximation$ of $B=\{b(v), v \in V\}$ by using the technique introduced in section “Rademacher averages”. CBCA can be divided into two phases. In the first phase, the CBCA algorithm generates the required number of high-quality centroids (lines 1–3). In the second phase, it describes the main procedure of how the algorithm approximates the betweenness centrality (lines 4–18).

We start with the description of the first phase.

- In line 1, the diameter of the graph is determined by using the procedure getDiameter(*G*) with the diameter method described in section “Centroids screening strategy”.
- In line 2, the number of required *t* centroids are obtained by following the formula (13) for calculating the centroids described in section “Centroids screening strategy”.
- In line 3, the CBCA algorithm obtains the high-quality network centroids based on the adjacency information entropy of section “Centroids quality screening strategy”, for which the shortest paths approximation described in section “Subgraphs construction for the shortest paths approximation” is used to determine the *t* subgraphs divisions and completes the approximation using the procedure getZone(*t*).

We now proceed to the second phase of the algorithm.

- In line 4 and line 5, the initial value is assigned to enable that $S_i$, which stores the sample size, is the empty set and *i* is the index iteration, while $m_i$ can guarantee the size of the sample $S_i$ considered at the $i^{th}$ iteration. Here $m_i \ge m_1$, which can guarantee that with probability $1-\frac{\delta }{2}$ to satisfy the $\varepsilon -approximation$. Then $\lambda$ is initialized as the null matrix by the CBCA algorithm, and the main task is to calculate the *k*-MCERA for each iteration (see section “Rademacher averages”).
- In each iteration of the for loop, our CBCA algorithm performs the following operations: First of all, in line 6, the desired pairs of sample nodes, obeying a uniform distribution, can be easily obtained by using the program getUniformSample(*P*) through the domain *P*.
- In line 7, At the $i^{th}$ iteration, it generates new samples $m_i-m_{i-1}$ and adds them to $S_{i-1}$ to obtain the new sample set $S_i$.
- In line 8, an upper bound on the number of samples required for approximation is calculated with the function effectiveSampleSchedule $(S_i)$, which is used to ensure that any sample *S* has sufficient sample size to satisfy the $\varepsilon -approximation$ with at least $1-\delta$ probability. Although any schedule can be used, we give a suitable sample schedule to satisfy the $\varepsilon -approximation$ in subsequent experiments.
- In line 9, we use the procedure updateValues $(s_i)$ to obtain the sample set *Q*. The set *Q* helps to compute the variance and Rademacher values needed in the subsequent procedure. $Q=\{(w, f_w(s_i)) for each w \in V s.t. f_w(s_i) \ne 0\}$. Then we extend to $\lambda$ to add $m_i-m_{i-1}$ columns, each column consists of *k* rows, so $\lambda \in \{0, 1\}^{k \times m_i}$ to facilitate the computation of samples of size *S*.
- In line 10, the $k \times m_i$ matrices are sampled by the getSampleRademacher procedure, where each matrix is a Rademacher r.v. (see section “Rademacher averages”).
- In line 11, the procedure getUpdate$(m_i,\lambda ,f_w(s_i))$ is used, which updates the value of the computed *k*-MCERA (see section “Rademacher averages”). And it can estimate $\tilde{B}$ (in line 12), where $\tilde{B}$ is a set of pairs $(v,\tilde{b}(v))$ for each $v \in V$, where $\tilde{b}(v)$ is the estimate of *b*(*v*). The value of $\varepsilon$ is computed (in line 13), and the computed value of $\varepsilon$ is large than the user-specified value of ${\bar{\varepsilon }}$. If the value of $\varepsilon$ is over the size of the user-specified value of ${\bar{\varepsilon }}$, then the iteration is finally stopped and $(\tilde{B},\varepsilon )$ is output (in lines 14-18).

## Experiments and analysis of results

In this section, we successfully implemented the CBCA algorithm and gave experimental evaluation results. We performed the experimental analysis in five aspects: sample size, running time, accuracy, different network characteristics, and dynamic centroids updating. We compared the sample size and running time of the CBCA algorithm with those of the algorithms Silvan and *rk*; and the accuracy rate with those of *bp* and *rk*. This is because Silvan is the newest algorithm and consistently outperforms other algorithms in terms of sample size and running time, while the *bp* algorithm has the highest accuracy rate among the known algorithms. We also tested five artificially generated random networks and networks with power-law property and Small-world property for network feature comparison. Finally, we gave the theoretical analysis and experimental results of dynamic centroids updating. All the above algorithms are guaranteed to satisfy $\varepsilon -approximation$ with $1-\delta$ probability for all nodes.

*Implementation and environment.* This part of the experiment is built on a Linux system, using Ubuntu 18.04 as the main operating system of the experiment platform, with hardware specifications: Intel(R) Core (TM) i9-9900K CPU@3.6GHz processor with 16GB of physical memory. It also has an NVIDIA GeForce RTX 2080Ti graphics processor with 11GB video memory capacity. Software drivers: The 2080Ti graphics processor comes with a 470.103.01 driver with CUDA 11.4 acceleration platform. All code is compiled via GCC 8.

*Dataset and parameters.* We used the publicly available real dataset SNAP from Stanford University. All features of the graphs are given in Table 2. Regarding the choice of multiple parameters, we first considered $\varepsilon \in \{0.01,0.006,0.0015,0.008,0.003\}$ for each graph. For $\delta$, we set its value to a fixed 0.1, and we tested different values of it separately in our experiments. We found that it has very little effect on the results due to the use of probabilistic tail bound perception leading to exponential dependence, which differs very little from that described by *rk*. We set the initial sample size $m_1$ to the case $R({\mathscr {F}},m)=0$, because this is the upper limit of the initial sample size that can be guaranteed with a high-quality approximation. The progressive sampling used was set to $m_i=1.5m_{i-1}$ in the CBCA algorithm concerning the sample schedule. For the parameter *k*-Monte Carlo trials, referring to Bavarian [18] and MCRapper [44]. It was fixed to 25, which shows that clear bounds can be obtained even using a small number of Monte Carlo trials, but of course, we also experimented with $k=50,100$, and 200 and found that it is not better than the case of $k=25$. We ran all the algorithms 5 times and reported $Avg \pm stddev$, which is representative of the standard deviation of a single measurement.

**Table 2:** 9 graphs, where *D* is the diameter of the graph (the longest one shortest path); *t* represents the number of prime centers; type *A* indicates a directed graph and *B* indicates an undirected graph.

G	V	E	Type	D	t
Wiki_Talk	2,394,385	5,021,410	A	9	2
p2p_Gnutella31	62,586	147,892	A	11	2
Ca-GrQc	5,242	14,496	B	17	3
Com-youtube	1,134,890	2,987,624	B	20	3
cit-HepPh	34,546	421,578	A	12	2
cit-HepTh	27,770	352,807	A	13	3
ca-AstroPh	18,772	198,110	B	14	3
Wiki_topcats	1,791,489	28,511,807	A	9	2
Soc-Epinions1	75,879	508,837	A	14	3

### Sample size

**Figure 3:**
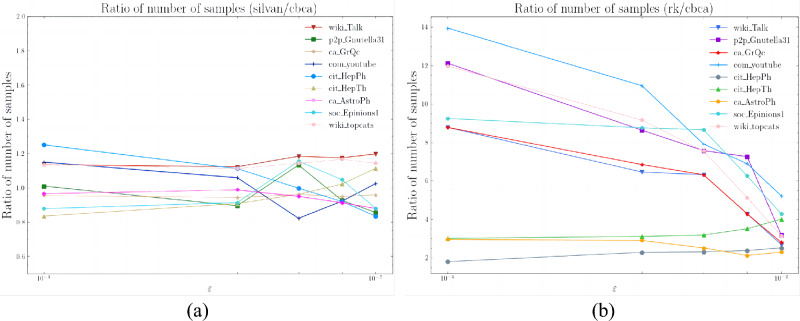
The ratio of the number of samples that can satisfy the high quality $\varepsilon -approximation$. (**a**) the ratio of the number of samples between Silvan and CBCA. (**b**) The ratio of the number of samples between *rk* and CBCA.

We first show a comparison figure of the required sample sizes on different datasets in Fig. 3.

Figure 3a illustrates the ratio of sample size required by Silvan and CBCA to achieve high-quality approximation. First, for the large graphs Wiki_Talk and Wiki_topcats, CBCA requires at least 10.3% and at most 19.7% less sample size than Silvan, respectively. Although both Silvan and CBCA algorithms employ the *k*-MCERA technique, CBCA adopts the shortest paths approximation, which can effectively reduce the time to compute the shortest paths and thus decrease the sample size. In some small graphs, such as ca-GrQc, Silvan’s sample size is smaller than CBCA’s, which is due to CBCA’s consideration of the graph diameter, while Silvan uses an empirical peeling technique to reduce the required sample size. Finally, the CBCA algorithm performs better on large graphs, which is consistent with our experimental objective.

As shown in Fig. 3b, the CBCA algorithm requires much smaller sample sizes than the *rk* algorithm. The difference is up to an order of magnitude lower; and at least several times smaller. This is closely related to the different methods they use to reduce the sample sizes. The *rk* algorithm relies on the VC dimension, which can guarantee a high-quality approximation, but it only considers the diameter of the graph and ignores other features. This leads to an overly conservative estimation of the sample size. However, our CBCA algorithm employs the state-of-the-art *k*-Monto Carlo trials technique to obtain the maximum correlation index between the nodes with sharp variance-aware probability tail bounds. This can effectively reduce the sample size and provide better guarantees.

In conclusion, the CBCA algorithm can better obtain the minimum number of samples required to satisfy the high-quality approximation, which illustrates the importance of our shortest paths approximation and *k*-Monto Carlo trials techniques.

### Running time

**Figure 4:**
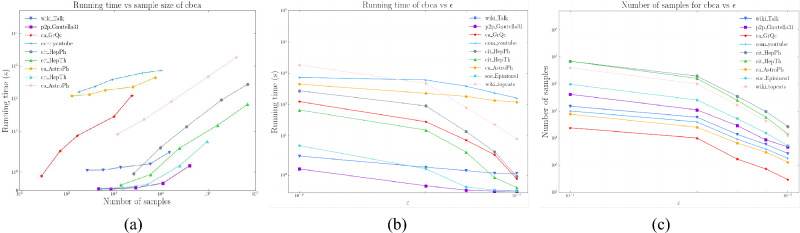
(**a**) Running time versus sample size. (**b**) Running time of CBCA versus $\varepsilon$. (**c**) Number of samples for CBCA versus $\varepsilon$.

**Figure 5:**
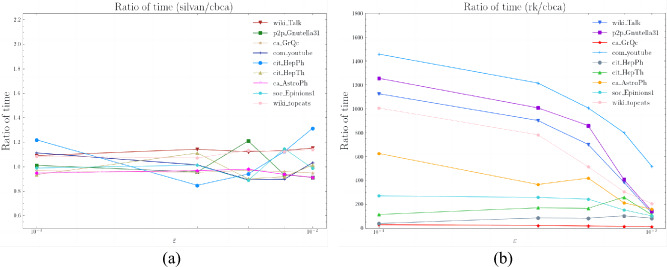
(**a**) The ratio of time between *rk* and CBCA. (**b**) The ratio of time between Silvan and CBCA.

we report the running time, sample size, and $\varepsilon$ of the CBCA algorithm on different datasets in Fig. 4. And we show a comparison of the required run times on different datasets in Fig. 5.

In Fig. 4, we can observe that the running time increases linearly with the sample size, because the main time-consuming step of the algorithm is sampling, which accounts for more than 90% of the running time. Similarly, from Fig. 4b,c, both the number of samples and the running time decrease as the error $\varepsilon$ increases, because a larger error $\varepsilon$ reduces the number of samples required to obtain a more accurate value. This also demonstrates that our algorithm does not waste time on calculating the centroids, but focuses on the useful work.

We can see the ratio of time required to achieve approximation in Fig. 5a. For the large graph Wiki_Talk and Wiki_topcats, CBCA performs better than Silvan in different errors $\varepsilon$, saving 7.1% to 15.2% of time. This is because the algorithm spends more than 90% of the time on sampling. Our CBCA algorithm uses the shortest path approximation, which can speed up the sampling time, while Silvan uses bidirectional BFS traversal, which leads to an increase in sampling time. When the graph is small, such as ca-GrQc, Silvan’s sampling speed is faster than CBCA’s. The results show that CBCA’s running time is faster in large graphs.

As shown in Fig. 5b, the CBCA algorithm can be two to three orders of magnitude faster than *rk* in large graphs and one order of magnitude faster even in small graphs. This is attributed to the improvement of CBCA based on the shortest paths approximation, reducing the running time, as well as the sharply wimpy variance technique for satisfying approximations of high quality, reducing the number of samples.

### Accuracy

**Figure 6:**
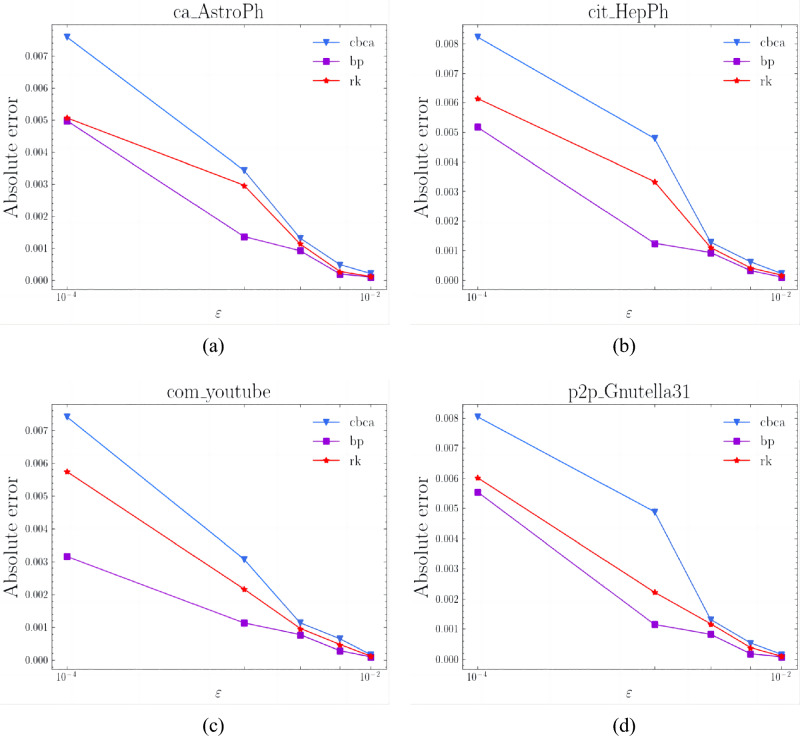
The absolute error of our CBCA algorithm in 4 real graphs. (**a**) ca_AstroPh. (**b**) cit_HepPh. (**c**) com_youtube. (**d**) p2p_Gnutella31.

we show a comparison of the absolute errors on the four datasets in Fig. 6.

In this section, we discuss the accuracy of the algorithm that we introduced in section “Preliminaries” of this paper. As shown in Fig. 6a–d, the CBCA algorithm can always guarantee that all nodes satisfy $\varepsilon -approximation$ with probability $1-\delta$. Moreover, the absolute error computed is smaller than $\varepsilon$, even by an order of magnitude. This indicates that the algorithm can perform better than the theoretical guarantee. This is due to the use of the technique of the *k*-Monto-Carlo trial, which can capture the probability tail bound with sharply wimpy variance, making a better balance between sample size and accuracy.

Compared with CBCA, the errors of both *bp* and *rk* algorithms are smaller. This is because the VC dimensional theory used by *rk* only considers the diameter of the graph without a more detailed understanding of the other structural underlying distributions of the graph. This results in too many samples, making it better in terms of accuracy guarantees. Moreover, the error of *bp* is minimal because the algorithm uses Hoeffding’s inequality and union bound. Although both algorithms can satisfy $\varepsilon -approximation$ with a low error and a probability of $1-\delta$, they consume a lot of time in respect of running time. Increasing the sample size of the number of samples to improve the accuracy sacrifices the running time.

### Different types of networks

**Figure 7:**
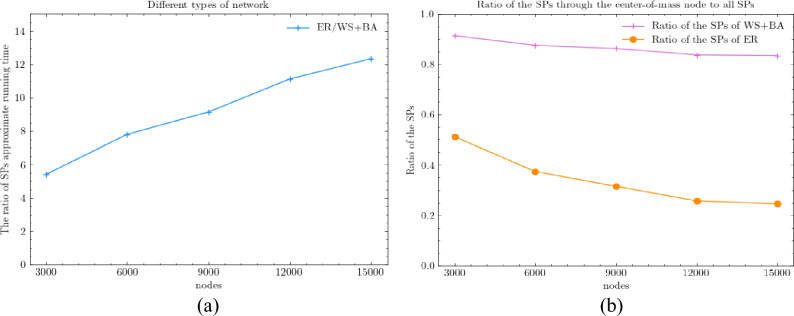
(**a**) The ratio of shortest paths approximation times for ER random networks to WS small-world networks and BA scale-free networks. (**b**) The ratio of shortest paths through the centroids to all shortest paths in ER random networks to WS small-world networks and BA scale-free networks.

We generate ER random networks^65^, WS small-world networks, and BA scale-free networks with 3000, 6000, 9000, 12000, and 15000 nodes (i.e., five graphs with random network properties and five graphs with the small-world property as well as power-law property, respectively).

Our Shortest Paths Approximation theory is based on two properties of complex networks: the small-world property and the power-law property. To verify this theory, we apply the shortest paths approximation to a random network. The clustering coefficient of random networks is small, while the clustering coefficient of small-world networks is large. Random networks do not have power-law distributions, while scale-free networks have a few high degree nodes and a large number of low degree nodes. These differences affect the effectiveness of the shortest paths approximation.

Figure 7a shows that the approximation time of the random network is at least 5.4 times longer than that of the small-world and scale-free network when the shortest paths approximation is used for all networks.

Figure 7b compares the ratio of shortest paths that pass through the selected centroids in the two types of networks. The figure shows that the shortest paths that pass through the centroids account for more than 81.3% of all shortest paths in the network with small-world and scale-free features, while in the random network, this ratio is only up to 52.1%.

This demonstrates the superiority of the CBCA algorithm’s shortest paths approximation in networks with small-world property and power-law property.

### Updating centroids in dynamic networks

According to section “Network Density and Clustering Coefficient”, we choose the network density and clustering coefficient to measure the dynamic changes of the network. Then, we establish a formula to represent the dynamic change before it occurs:

27 $$\begin{aligned} \begin{aligned} f_{ea}=\phi {\bar{C}} +(1-\phi )d_{G}\,, \end{aligned} \end{aligned}$$

for $\phi -$value picking $0,\frac{1}{4},\frac{1}{3},\frac{1}{2},\frac{2}{3},\frac{3}{4},$ and 1, respectively. 7 plans are selected.

We conduct comparative experiments on different methods or parameters, and based on the evaluation criteria of accuracy and efficiency, we choose:

28 $$\begin{aligned} \begin{aligned} f_{ea}=\frac{1}{3}{\bar{C}}+\frac{2}{3}d_{G}. \end{aligned} \end{aligned}$$

Therefore, by setting different thresholds in the networks with small-world and power-law characteristics generated in section “Different types of networks”, and recalculating the centroids. It is judged that the recalculation of the centroid nodes should be performed in the case of exceeding a certain threshold.

The initial state network is characterized by $f_{ea_1}=\frac{1}{3}\bar{C_1}+\frac{2}{3}d_{G1}$, and after the dynamic updating, it is $f_{ea_2}=\frac{1}{3}\bar{C_2}+\frac{2}{3}d_{G2}$.

Threshold qualifier:

29 $$\begin{aligned} \begin{aligned} |\frac{f_{ea_2}-f_{ea_1}}{f_{ea_1}}|\in \{0,1\} \>THV\,, \end{aligned} \end{aligned}$$

we proceed to set the resulting THV for the threshold qualifier to 0.2, 0.3, 0.4, 0.5, and 0.6.

**Table 3:** The centroids are to be updated at different thresholds. *U* means the centroids do not need to be updated, and *N* means it needs to be updated.

Graph	t	0.2	0.3	0.4	0.5	0.6
3000	3	U	U	U	U	U
6000	3	U	U	U	U	U
9000	2	U	U	U	N	N
12000	3	U	U	N	N	N
15000	2	U	N	N	N	N

It can be seen from Table 3 that the original centroids need to be updated when the threshold THV exceeds 0.5. This shows that our CBCA algorithm updates the centroids only if the THV is set to a value greater than 0.5, saving a lot of time.

## Conclusion

In this paper, we present a novel betweenness centrality approximation algorithm based on progressive sampling and shortest paths approximation. The algorithm firstly uses the adjacency information entropy to generate network centroids and constructs an efficient shortest paths approximation strategy; then, it uses the *k*-Monto Carlo trials technique to trade off the sample size and error to obtain a high-quality approximation of the betweenness centrality of all nodes. The algorithm can also handle dynamic networks with frequent BC changes by using a centroid updating strategy based on network density and clustering coefficients.

Our experimental results show that our algorithm outperforms the baseline algorithm for the same probability in various networks. Our algorithm can efficiently output high-quality approximations of the node betweenness centrality in large-scale complex networks. Our algorithm can also be applied to network analysis and applications, such as identifying the most influential or central nodes in a network, which can help us understand the network structure and function, as well as optimize its performance or resilience. However, our algorithm also has some limitations and challenges, such as relying on the quality of the network centroids, which may not always be optimal or representative. Therefore, we intend to extend our algorithm to address these issues in future work, such as exploring different methods or criteria to select or update network centroids, applying our algorithm to different types of networks, and applying our algorithm to other network analysis tasks.

Moreover, the network centroids and shortest paths approximation methods proposed in this paper can also be tried for other centrality measures, such as degree centrality, closeness centrality, etc. These centrality measures can also reflect the importance or influence of network nodes in different aspects, and have many practical applications. We believe that our methods can provide an effective and flexible approximation strategy for other centrality measures, and can adapt to different scales and characteristics of networks. We also believe that the methods proposed in this paper can be used to improve the speed and quality of data analysis and mining, such as finding similar or different points or groups in data faster, evaluating important or abnormal points or edges in data more accurately, etc. These are very important and valuable problems in the field of data science, and have many practical applications.

### Supplementary Information

Supplementary Information.

## Data Availability

The data generated and analysed during the current study are available in the OSFHOME repository: https://osf.io/xhs2q/?view_only=eed9bb3e28ac48f4acd527117e2037f4.
