# Supplementary material for: Estimation and update of betweenness centrality with progressive algorithm and shortest paths approximation
Source: Sci Rep. 2023 Oct 10;13:17110. doi: 10.1038/s41598-023-44392-0 (PMC10564764; doi:10.1038/s41598-023-44392-0)
Supplement: Supplementary file 1 — Supplementary Information. [file 41598_2023_44392_MOESM1_ESM.pdf]

Supplementary Material

Appendix A.1

We prove Theorem 2.1.

Theorem 2.1:

Let $\mathscr{F}$ be a family of functions from domain P to [0,1], for each $f \in \mathscr{F}$ taken the probability uniform distribution μ over the P, it holds:

$$var = \sup_{f\in\mathscr{F}} E_\mu[f^2]. \tag{1}$$

Proof: Due to the normalization factor of $\frac{1}{n(n-1)}$, the value of BC is almost zero, especially on large-scale networks[1]. It is reasonable to be considered:

$$var = E_\mu[f^2] - \{E(f)\}^2 \le E_\mu[f^2].$$

Appendix A.2

We are now ready to prove Theorem 2.2. Our most significant technical contributions are in this paper.

Theorem 2.2:

For $k,m \ge 1$ and the function $f \in \mathscr{F}$, where $\mathscr{F}$ be a family of functions from P to [0,1]. Let $\lambda \in \{-1,+1\}^{k\times m}$ be an $k\times m$ matrix of Rademacher random variables, so that $\lambda \in \{-1,+1\}$ independently and with equal probability $\frac{1}{2}$. Let S be a sample size of m drawn i.i.d. from P, taken a distribution μ. For each $\delta \in (0,1)$, define:

$$\begin{aligned} V(f) &\doteq \alpha + \frac{ln\frac{3}{\delta}}{m} + \sqrt{(\frac{ln\frac{3}{\delta}}{m})^2 + \frac{2\alpha ln\frac{3}{\delta}}{m}} \\ \tilde{R}(\mathscr{F},S) &\doteq \tilde{R}^k_m(\mathscr{F},S,\sigma) + \frac{2ln\frac{3}{\delta}}{km} + \sqrt{(\frac{2ln\frac{3}{\delta}}{km})^2 + \frac{4(\tilde{R}^k_m(\mathscr{F},S,\lambda)+\alpha)ln\frac{3}{\delta}}{km}} \\ R(\mathscr{F},m) &\doteq \tilde{R}(\mathscr{F},S) + \frac{ln\frac{3}{\delta}}{m} + \sqrt{(\frac{ln\frac{3}{\delta}}{m})^2 + \frac{2\tilde{R}(\mathscr{F},S)ln\frac{3}{\delta}}{m}} \\ \varepsilon &\doteq 2R(\mathscr{F},m) + \frac{ln\frac{3}{\delta}}{3m} + \sqrt{(\frac{ln\frac{3}{\delta}}{3m})^2 + \frac{2R(\mathscr{F},m)ln\frac{3}{\delta}}{m}}, \end{aligned} \tag{2}$$

With the probability at least $1-\delta$ over the choice of S and λ.

We first prove the origin of each formula in four steps.

Step 1:

Theorem A.2.1 (Thm.7.5.8[2]) :

With probability $\ge 1-\eta$ over S, it holds:

$$\sup_{f\in\mathscr{F}} E(f^2) \le \alpha + \frac{ln\frac{1}{\eta}}{m} + \sqrt{(\frac{ln\frac{1}{\delta}}{m})^2 + \frac{2\alpha ln\frac{1}{\eta}}{m}}.$$

Proof: From Theorem 2.1, $V(f) = \sup_{f\in\mathscr{F}} E(f^2)$, thus replacing $\frac{1}{\lambda}$ with $\frac{3}{\delta}$, we can obtain the result.

Step 2:

Before proving that $\tilde{R}(\mathscr{F},S) \doteq \tilde{R}^k_m(\mathscr{F},S,\lambda) + \frac{2ln\frac{3}{\delta}}{nm} + \sqrt{(\frac{2ln\frac{3}{\delta}}{nm})^2 + \frac{4(\tilde{R}^n_m(\mathscr{F},S,\lambda)+\alpha)ln\frac{3}{\delta}}{nm}}$, we need to define two functions that we need to use: the self-boundary function and $g_{j,i}(\sigma)$.

Definition 1: (φ,γ)-self-bounding function

Let $X = (X_1,...,X_n)$ be a vector of random variables X_i, each taking values in a measurable set χ and let g: χ^n map R be a non-negative measurable function. The denote g_i a function from χ^{n-1} map R.

A function g is a (φ,γ)-self-bounding function, for each $X \in \chi^n$:

$$0 \leq g(X) - g_i(X^{(i)}) \leq 1$$
$$\sum_{i=1}^{n} \{g(X) - g_i(X^i)\} \leq \phi g(X) + \gamma, \tag{3}$$

where $X^{(i)} = (X_1, ..., X_{i-1}, X_{i+1}, ..., X_N) \in \chi^{n-1}$.

Theorem A.2.2:

Let $\sigma \in \{-1,+1\}^{n\times m}$ be a n×m matrix, define the function of $g(\sigma)$:

$$g(\sigma) \doteq nm\tilde{R}_m^n(\mathscr{F}, m, \sigma), \tag{4}$$

$g(\sigma)$ is a self-bounding function of $(1, 2nm\alpha)$. Where $\alpha = \sup_{f\in\mathscr{F}} \frac{1}{m}\sum_{i=1}^{m}(f(s_i))^2$.

Definition 2: the function of $g_{j,i}(\sigma)$, for $j \in [1,n]\, and\, i \in [1,m]$ is defined as:

$$g_{j,i}(\sigma) \doteq \inf_{\sigma'_{j,i}\in\{-1,+1\}} \left\{ \sum_{q=1,q\neq j}^{n} \left[\sup_{f\in\mathscr{F}} \sum_{q=1}^{m} f(s_z)\sigma_{q,z} \right] + \sup_{f\in\mathscr{F}} \left\{ \sum_{z=1,z\neq i} (\sigma_{j,z} f(s_z)) + (\sigma'_{j,i} f(s_i)) \right\} \right\},$$

we denote by $g(\sigma)$ the function that replaces the element $\sigma_{j,i}$ at the position (i,j) of σ with σ', and we take the smallest value over σ'.

Proof of Theorem A.2.2:

Now proceed to prove Theorem A.2.2, i.e., prove that:

$$0 \leq g(\sigma) - g_{j,i}(\sigma) \leq 1$$
$$\sum_{j=1}^{n}\sum_{i=1}^{m}(g(\sigma) - g_{j,i}(\sigma))^2 \leq \phi g(\sigma) + \gamma, (\phi, \gamma) \geq 0.$$

First, we can rewrite $g(\sigma)$ as the following:

$$g_{j,i}(\sigma) = \min\left[\sum_{q=1,q\neq j}^{n} \left[\sup_{f\in\mathscr{F}} \sum_{z=1}^{m} \sigma_{q,z} f(s_z) \right] + \sup_{f\in\mathscr{F}} \left\{ \sum_{z=1,z\neq i}^{m} (\sigma_{j,z} f(s_z) - f(s_i)) \right\}, \sum_{q=1,q\neq j}^{n} \left[\sup_{f\in\mathscr{F}} \sum_{z=1}^{m} \sigma_{q,z} f(s_z) \right] + \sup_{f\in\mathscr{F}} \left\{ \sum_{z=1,z\neq i}^{m} (\sigma_{j,z} f(s_z) + f(s_i)) \right\} \right].$$

It is easy to see that at least one element of this min equation is equal to $g(\sigma)$, so the minimum is either $g(\sigma)$ or smaller than $g(\sigma)$. Next, we start the proof of Theorem A.2.2 that:

$$\begin{aligned} g_{j,i}(\sigma) &\doteq \inf_{\sigma'_{j,i}} \sum_{q=1,q\neq j}^{n} \left[\sup_{f\in\mathscr{F}} \sum_{z=1}^{m} \sigma_{q,z} f(s_z) \right] + \sup_{f\in\mathscr{F}} \left\{ \sum_{z=1,z\neq i}^{m} (\sigma_{j,z} f(s_z) + \sigma'_{j,i} f(s_i))) \right\} \\ &= \sum_{q=1,q\neq j}^{n} \left[\sup_{f\in\mathscr{F}} \sum_{z=1}^{m} \sigma_{q,z} f(s_z) \right] + \inf_{\sigma'_{j,i}} \left\{ \sup_{f\in\mathscr{F}} \left\{ \sum_{z=1,z\neq i}^{m} (\sigma_{j,i} f(s_z)) + \sigma'_{j,i} f(s_i) \right\} \right\} \\ &\geq \sum_{q=1,q\neq j}^{n} \left[\sup_{f\in\mathscr{F}} \sum_{z=1}^{m} \sigma_{q,z} f(s_z) \right] + \sup_{f\in\mathscr{F}} \left\{ \inf_{\sigma'_{j,i}\in\{-1,+1\}} \left\{ \sum_{z=1,z\neq i}^{m} (\sigma_{j,i} f(s_z)) + \sigma'_{j,i} f(s_i) \right\} \right\} \\ &= \sum_{q=1,q\neq j}^{n} \left[\sup_{f\in\mathscr{F}} \sum_{z=1}^{m} \sigma_{q,z} f(s_z) \right] + \sup_{f\in\mathscr{F}} \left\{ \sum_{z=1,z\neq i}^{m} (\sigma_{j,i} f(s_z)) + \inf_{\sigma'_{j,i}\in\{-1,+1\}} \{\sigma'_{j,i} f(s_i)\} \right\}. \end{aligned}$$

For a given σ, let $f_j^{\star}$ be one of the functions of $\mathscr{F}$ attaining the supremum of $\sup_{f\in\mathscr{F}} \sum_{z=1}^{m} \sigma_{j,i} f(s_z)$. Thus, We can keep writing this up here:

$$
\begin{aligned}
g_{j,i}(\sigma) &\geq \sum_{q=1,q\neq j}^{n}\left[\sup_{f\in\mathscr{F}}\sum_{z=1}^{m}\sigma_{q,z}f(s_z)\right] + \sup_{f\in\mathscr{F}}\left\{\sum_{z=1,z\neq i}^{m}(\sigma_{j,i}f(s_z)) + \inf_{\sigma'_{j,i}\in\{-1,+1\}}\{\sigma'_{j,i}f(s_i)\}\right\} \\
&\geq \sum_{q=1,q\neq j}^{n}\left[\sup_{f\in\mathscr{F}}\sum_{z=1}^{m}\sigma_{q,z}f(s_z)\right] + \sum_{z=1,z\neq i}^{m}(\sigma_{j,i}f_j^{\star}(s_z)) + \inf_{\sigma'_{j,i}\in\{-1,+1\}}\{\sigma'_{j,i}f_j^{\star}(s_i)\} \\
&= \sum_{q=1,q\neq j}^{n}\left[\sup_{f\in\mathscr{F}}\sum_{z=1}^{m}\sigma_{q,z}f(s_z)\right] + \sum_{z=1,z\neq i}^{m}(\sigma_{j,i}f_j^{\star}(s_z)) + \sigma_{j,i}f_j^{\star}(s_i) - \sigma_{j,i}f_j^{\star}(s_i) \\
&+ \inf_{\sigma'_{j,i}\in\{-1,+1\}}\{\sigma'_{j,i}f_j^{\star}(s_i)\} \\
&= g(\sigma) - \sigma_{j,i}f_j^{\star}(s_i) + \inf_{\sigma'_{j,i}\in\{-1,+1\}}\{\sigma'_{j,i}f_j^{\star}(s_i)\},
\end{aligned}
$$

where $f_j^{\star}(s_i)\in[0,1]$, we can obtain that: $g_{j,i}(\sigma)\geq g(\sigma)-\sigma_{j,i}f_j^{\star}(s_i)-|f_j^{\star}(s_i)|\geq g(\sigma)-1$.

Now, the proof of $\phi=1,\gamma=2nm$ as follows:

$$
\begin{aligned}
\sum_{j=1}^{n}\sum_{i=1}^{m}\Big(g(\sigma)-g_{j,i}(\sigma)\Big)^2 &\leq \sum_{j=1}^{n}\sum_{i=1}^{m}\Big(\sigma_{j,i}f_j^{\star}(s_i)+|f_j^{\star}(s_i)|\Big)^2 \\
&= \sum_{j=1}^{n}\sum_{i=1}^{m}\Big([\sigma_{j,i}f_j^{\star}(s_i)]^2+|f_j^{\star}(s_i)|^2+2\sigma_{j,i}f_j^{\star}(s_i)|f_j^{\star}(s_i)|\Big) \\
&= \sum_{j=1}^{n}\sum_{i=1}^{m}\Big(2f_j^{\star}(s_i)^2+2\sigma_{j,i}f_j^{\star}(s_i)|f_j^{\star}(s_i)|\Big) \\
&\leq \sum_{j=1}^{n}\sum_{i=1}^{m}\sigma_{j,i}f_j^{\star}(s_i)+2\sum_{j=1}^{n}\sum_{i=1}^{m}f_j^{\star}(s_i)^2 \\
&= g(\sigma)+2\sum_{j=1}^{n}\sum_{i=1}^{m}f_j^{\star}(s_i)^2 \\
&\leq g(\sigma)+2n\sup_{f\in\mathscr{F}}\sum_{i=1}^{m}(f(s_i))^2 \\
&= g(\sigma)+2nm\alpha,
\end{aligned}
$$

obtaining the statement.

Theorem A.2.3[3]

let $\lambda\in\{-1,+1\}^{n\times m}$ be an $n\times m$ matrix of matrix of Rademcher random variables, $\lambda_{j,i}\in\{-1,+1\}$ with probability $\frac{1}{2}$ taken each one and independent. Then, for all $0\leq\tau\leq\tilde{R}(\mathscr{F},S)$:

$$
Pr(\tilde{R}(\mathscr{F},S)\geq\tilde{R}_m^n(\mathscr{F},S,\lambda)+\tau)\leq exp(-\frac{nm\tau^2}{4(\check{R}(\mathscr{F},S)+\alpha)}). \tag{5}
$$

Theorem A.2.3 plays a role in solving the second formula, so we prove it as follows:

The function of f, defines:

$$
\hat{\mathscr{F}}\doteq\hat{f}(x)\doteq\frac{f(x)}{2}:f\in\mathscr{F},\forall x\in\chi.
$$

With theorem A.2.2, we know that $g(\sigma)=nm\tilde{R}_m^n(\hat{F},S,\lambda)$ is a $1,2nm\sigma$ self-bounding function. It is easily to get the statement that $E_\lambda[nm\tilde{R}_m^n(\hat{\mathscr{F}},S,\lambda)]=nm\tilde{R}(\hat{\mathscr{F}},S)$. We apply (Theorem.7[3]), obtaining:

$$Pr(nm\tilde{R}(\hat{\mathscr{F}},S) \geq nm\tilde{R}_m^n(\hat{\mathscr{F}},S,\lambda)+t) \leq exp(-\frac{t^2}{2(nm\tilde{R}(\hat{\mathscr{F}},S)+2nm\alpha)}), \tag{6}$$

we use the fact that $\tilde{R}(\hat{\mathscr{F}},S) = \frac{\tilde{R}(\mathscr{F},S)}{2}$, $\tilde{R}_m^n(\hat{\mathscr{F}},S,\lambda) = \frac{\check{R}_m^n(\mathscr{F},S,\lambda)}{2}$ and $\alpha_{\hat{\mathscr{F}}} = \frac{\alpha}{4}$.

It follows that:

$$Pr(\frac{nm}{2}\tilde{R}(\mathscr{F},S) \geq \frac{nm}{2}\tilde{R}_m^n(\mathscr{F},S,\lambda)+t \leq exp(-\frac{t^2}{nm(\tilde{R}(\mathscr{F},S))+\alpha})), \tag{7}$$

replacing t by $\frac{\tau nm}{2}$ achieve the Theorem A.2.3.

The proof of the key equation begins below:

$$\tilde{R}(\mathscr{F},S) = \tilde{R}_m^n(\mathscr{F},S,\lambda) + \frac{2ln\frac{1}{\delta}}{nm} + \sqrt{(\frac{2ln\frac{1}{\delta}}{nm})^2 + \frac{4(\tilde{R}_m^n(\mathscr{F},S,\lambda)+\alpha)ln\frac{1}{\delta}}{nm}}.$$

Proof: From Theorem A.2.3, we have the fact that, with probability $\geq 1-\delta$,

$$\tilde{R}(\mathscr{F},S) \leq \tilde{R}_m^n(\mathscr{F},S,\lambda) + \sqrt{\frac{4(\tilde{R}(\mathscr{F},S)+\alpha)ln\frac{1}{\delta}}{nm}}.$$

The bound of $\tilde{R}(\mathscr{F},S)$ can be obtained by the function of $b(x)$, which can find the fixed point.

$$b(x) \doteq \tilde{R}_m^n(\mathscr{F},S,\lambda) + \sqrt{\frac{4(x+\alpha)ln\frac{1}{\delta}}{nm}}.$$

Lemma 1. *let* $d, j, v \geq 0$. *The fixed point of*

$$b(x) \doteq d + \sqrt{j+vx},$$

is at

$$x \doteq d + \frac{v}{2} + \sqrt{\frac{v^2}{4}+j+vj}\cdot$$

Therefore, we apply lemma 1 to obtain the statement.

Step 3:

We have rigorously proved the two formulas before, and then our main technical proof Theorem 2.2 is based on the concentration inequality for Rademachaer Averages, for the supremum deviation $S(\mathscr{F},S) = \sup_{f\in\mathscr{F}} |\rho_s(f)-\rho_\mu(f)|$. To facilitate the discussion, we write $S(\mathscr{F},S)$ as Z[4].

We now start to describe. Then, define the *Rademacher complexity* $(RC)R(\mathscr{F},m)$ of a set of functions $\mathscr{F}$ as the expection of the ERA over S. $R(\mathscr{F},m) \doteq E_S[\tilde{R}(\mathscr{F},S)]$. The following central results correlate $\tilde{R}(\mathscr{F},m)$ with the expected supremum deviation.

Lemma 2. *Symmetrization lemma*[5]

$$E_S[Z] \leq 2R(\mathscr{F},m). \tag{8}$$

The following shows the deviation of the variance-dependent constraint above its expected value.

Theorem A.2.4[6] *Let* $Z = \sup_{f\in\mathscr{F}} |\rho_s(f)-\rho_\mu(f)|$. *Then, with probability at least* $1-\lambda$ *over* S, *it holds*

$$Z \le E[Z] + \frac{ln\frac{1}{\lambda}}{3m} + \sqrt{(\frac{ln\frac{1}{\lambda}}{3m})^2 + \frac{2(E[Z]+1)ln\frac{1}{\lambda}}{m}}.$$

We apply lemma 2. to Theorem A.2.3 can obtain

$$\varepsilon \doteq 2R(\mathscr{F},m) + \frac{ln\frac{1}{\lambda}}{3m} + \sqrt{(\frac{ln\frac{1}{\lambda}}{3m})^2 + \frac{2R(\mathscr{F},m)ln\frac{1}{\lambda}}{m}},$$

the next result bounds $R(\mathscr{F},m)$ above its estimated $\tilde{R}(\mathscr{F},S)$.

Step 4:

Theorem A.2.5.[7]

With probability $\ge 1-\lambda$ *over* S, *it holds*

$$R(\mathscr{F},m) \le \tilde{R}(\mathscr{F},S) + \frac{ln\frac{1}{\lambda}}{m} + \sqrt{(\frac{ln\frac{1}{\lambda}}{m})^2 + \frac{2\tilde{R}(\mathscr{F},S)ln\frac{1}{\lambda}}{m}}.$$

Conclusion:

Now we prove Theorem 2.2 in its entirety, the most important part in our paper.

Proof. In the 4 formulas for our most vital results, replace $\frac{3}{\delta}$ with $\frac{1}{\lambda}$ to obtain Theorem 2.2.

Appendix A.3

We now prove Theorem 3.1, which provides probabilistic quality assurance for the CBCA algorithm.

Theorem 3.1:

With probability at least $1-\delta$ *for the CBCA algorithm, the output* $(\tilde{B},\varepsilon)$, *such that* $|b(v)-\tilde{b}(v) \le \varepsilon|$.

Before proving, we need to understand the following facts will improve the efficiency of proof.

Facts:

(1). At the end of each iteration, the wimpy variance $V(f)$, for any $f \in \mathscr{F}$, can be computed by replacing δ with probability $\frac{\delta}{2^{i+1}}$ by the Eq.(2) in Theorem 2.2.

(2). At the end of each iteration, Monte Carlo empirical Rademacher, empirical Rademacher values, and Rademacher values, for any $fin\mathscr{F}$, can be computed by replacing δ with probability $\frac{\delta}{2^{i+1}}$ by the Eq.(2) in Theorem 2.2.

(3). At the end of each iteration, ε, for any $f \in \mathscr{F}$, can be computed by replacing δ with probability $\frac{\delta}{2^{i+1}}$ by the Eq.(2) in Theorem 2.2.

(4). After sampling m samples, the maximum error $S(\mathscr{F},S)$ is at most ε (i.e., $m_i \ge m_1$), and the probability is $1-\frac{\delta}{2}$. (this can refer to the Matteo[8])

(5). For each $i \ge 1, S_i = \{m_1,...,m_{s_i}\}$ is the set of S_i independent uniform samples from P.

The probability in Theorem 3.1 is taken on all realizations of sequence S_i, that is, on all realizations of sequence m_j.

Proof: Let events $E1$ and $E2$ be defined as:

$$E1 = \{\exists i \ge 1\, s.t.\, |b(v)-\tilde{b}(v)| > \varepsilon\}$$
$$E2 = \{\exists j,i \ge 1\, s.t.\, |b(v)-\tilde{b}(v)| > \varepsilon\}.$$

It can be known that the output of the algorithm satisfies the $\varepsilon-approximation$ condition when both events $E1$, $E2$ are wrong. Thus, we only need to prove $Pr(E1 \cup E2) \le \delta$.

For statement the sake of fact (4), we can obtain that $Pr(E1) \le \frac{\delta}{2}$.

In consideration of fact (1)(2)(3)(5), we can be calculated:

$$Pr(E2) = Pr(\exists j,i \quad s.t.\, |b(v)-\tilde{b}_{s_{i_j}}| > \varepsilon) \le \sum_i Pr(|b(v)-\tilde{b}_{s_{i_j}}| > \varepsilon)$$
$$\le \sum_i \frac{\delta}{2^{i+1}} \le \frac{\delta}{2}.$$

We can obtain that

$$Pr(E1 \cup E2) \leq Pr(E1) + Pr(E2) \leq \delta.$$

References

1. Cousins, C., C. Wohlgemuth & Riondato, M. Bavarian: Betweenness centrality approximation with variance-aware rademacher averages.acm transactions on knowledge discovery from data. *ACM Transactions on Knowl. Discov. from Data* **17(6)**, 1–47, DOI: 10.1145/3577021 (2023).
2. Pellegrina, L. & Vandin, F. Efficient mining of the most significant patterns with permutation testing. *Assoc. for Comput. Mach.* 2070–2079, DOI: 10.1145/3219819.3219997 (2018).
3. Pellegrina, L. Sharper convergence bounds of monte carlo rademacher averages through self-bounding functions. *arXiv e-prints* arXiv:2010.12103, DOI: 10.48550/arXiv.2010.12103 (2020).
4. Shalev-Shwartz, S. & Ben-David, S. Understanding machine learning: From theory to algorithms. *Camb. university press* (2014).
5. Mitzenmacher, M. & Upfal, E. Probability and computing: Randomization and probabilistic techniques in algorithms and data analysis. *Camb. university press* (2017).
6. Cousins, C. & Riondato, M. Sharp uniform convergence bounds through empirical centralization. *Adv. Neural Inf. Process. Syst.* **33**, 15123–15132 (2020).
7. Boucheron, G., S. Lugosi & Massart, P. Concentration inequalities: A nonasymptotic theory of independence. *Oxf. university press* (2013).
8. Riondato, M. & Kornaropoulos, E. M. Fast approximation of betweenness centrality through sampling. *Proc. 7th ACM international conference on Web search data mining* 413–422, DOI: 10.1145/2556195.2556224 (2014).
